# Physiological maturation and drug responses of human induced pluripotent stem cell-derived cortical neuronal networks in long-term culture

**DOI:** 10.1038/srep26181

**Published:** 2016-05-18

**Authors:** A. Odawara, H. Katoh, N. Matsuda, I. Suzuki

**Affiliations:** 1 Department of Electronics, Graduate School of Engineering, Tohoku Institute of Technology, 35-1 Yagiyama Kasumicho, Taihaku-ku, Sendai, Miyagi, 982-8577, Japan; 2 Japan Society for the Promotion of Science, 5-3-1 Koujimachi, Chiyoda-ku, Tokyo, 102-0083, Japan.

## Abstract

The functional network of human induced pluripotent stem cell (hiPSC)-derived neurons is a potentially powerful *in vitro* model for evaluating disease mechanisms and drug responses. However, the culture time required for the full functional maturation of individual neurons and networks is uncertain. We investigated the development of spontaneous electrophysiological activity and pharmacological responses for over 1 year in culture using multi-electrode arrays (MEAs). The complete maturation of spontaneous firing, evoked responses, and modulation of activity by glutamatergic and GABAergic receptor antagonists/agonists required 20–30 weeks. At this stage, neural networks also demonstrated epileptiform synchronized burst firing (SBF) in response to pro-convulsants and SBF suppression using clinical anti-epilepsy drugs. Our results reveal the feasibility of long-term MEA measurements from hiPSC-derived neuronal networks *in vitro* for mechanistic analyses and drug screening. However, developmental changes in electrophysiological and pharmacological properties indicate the necessity for the international standardization of culture and evaluation procedures.

Human neurons and neural networks generated from human induced pluripotent stem cells (hiPSCs) and embryonic stem (ES) cells hold great potential for drug discovery, toxicity testing, elucidating disease mechanisms, and regenerative medicine^1^,^2^,^3^,^4^,^5^. Over the past decade, hiPSC-derived neurons from multiple brain regions have been isolated and characterized for disease modeling and drug screening^3^,^6^,^7^,^8^. Indeed, many researchers have turned to stem cell systems because of their pluripotency for differentiation into specific human cell types. For instance, a recent study used hiPSC-derived dopaminergic neurons to screen a group of compounds for neuroprotective efficacy against early-stage Parkinson’s disease^9^.

However, the functional immaturity of human iPSC-derived neurons in short-term culture and lack of standard evaluation methods may limit the clinical translation of results. Several culture methods have been proposed to enhance the *in vitro* phenotypic maturation of human iPSC-derived neurons, such as co-culture with astrocytes. Our group and a few other researchers have previously demonstrated that co-culture with astrocytes facilitated the long-term *in vitro* survival and maturation of human neurons^10^,^11^,^12^. Because physiologically relevant assays are required to drive the discovery of safer, more efficacious medicines, it is critical that these cells and networks develop physiological and pharmacological properties closely resembling those of mature human neurons *in situ*.

Multi-electrode array (MEA) systems have proven useful for the non-invasive, real-time, multi-point measurement of cultured mammalian neuronal network and brain slice activity over long periods^13^,^14^,^15^,^16^,^17^,^18^. For example, MEAs have been successfully used to measure extracellular field potentials and drug responses from iPSC-derived cardiomyocytes for the screening of compounds with QT prolongation and proarrhythmic potential^19^,^20^, and a standard protocol for the MEA evaluation of cardiomyocytes is being established^21^. Our group reported the first measurements of pharmacological responses and activity-dependent plasticity from iPSC-derived neurons using an MEA system^10^. Although this suggests that MEA studies are suitable for the *in vitro* evaluation of iPSC-derived neuronal networks, no study has examined clinical drug responses against disease sequela such as neurodegeneration and epileptiform activity. To establish a drug evaluation assay for hiPSC-derived neuronal networks using MEA, it is first necessary to evaluate the culture conditions and duration necessary for functional maturation so that the model mimics as closely as possible the electrophysiological and pharmacological characteristics of human neuronal networks *in vivo*.

The cerebral cortex is the integrative and executive center of the mammalian central nervous system (CNS), making up over three quarters of human brain volume. Many CNS diseases are associated with the maldevelopment or degeneration of cortical neurons, such as epilepsy, autism, stroke, and Alzheimer’s disease. Therefore, the cerebral cortex is a major focus of drug screening studies. Synchronized burst firing (SBF) is a critical mechanism for development and information conduction under physiological conditions^22^,^23^,^24^,^25^, whereas uncontrolled SBFs is a hallmark of the epileptic brain. These SBFs are also generated in rat cortical neuronal networks *in vitro*, allowing for electrophysiological characterization and anticonvulsant screening. However, species differences warrant similar studies in human cortical circuits *in vitro*.

Here we characterized the changes in electrophysiological and pharmacological properties of individual hiPSC-derived cortical neurons and neuronal networks during long-term culture (up to 77 weeks) using MEAs. We also measured SBFs induced by a proconvulsant and suppressed by clinical anticonvulsants to demonstrate the utility of this culture model for the analysis of pathological mechanisms and drug discovery.

## Results

### Morphology of hiPSC-derived cortical neurons

**Figure 1:**
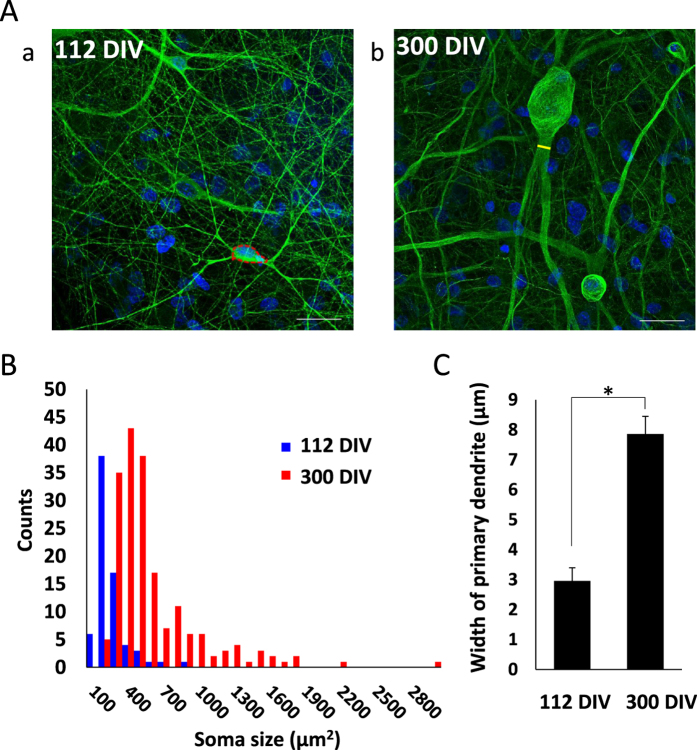
(**A**) Morphology of hiPSC-derived cortical neurons depends on days *in vitro* (DIV). (**A**) Fluorescent images of hiPSC-derived cortical neurons at 112 (a) and 300 DIV (b). Neurons were immunostained for β-tubulin III (green) and the nuclei counterstained with Hoechst 33258. Scale bars = 50 μm. Red dot circle in (a) shows the somal area and yellow line in (b) indicates the width of the primary dendrite (measured using ImageJ software). (**B**) Histogram of somal size for 112 and 300 DIV neurons. Bin size is 100 μm^2^. (n > 100) (**C**) Comparison of primary dendrite width between 112 and 300 DIV. (*P < 0.01, n > 100).

**Figure 2:**
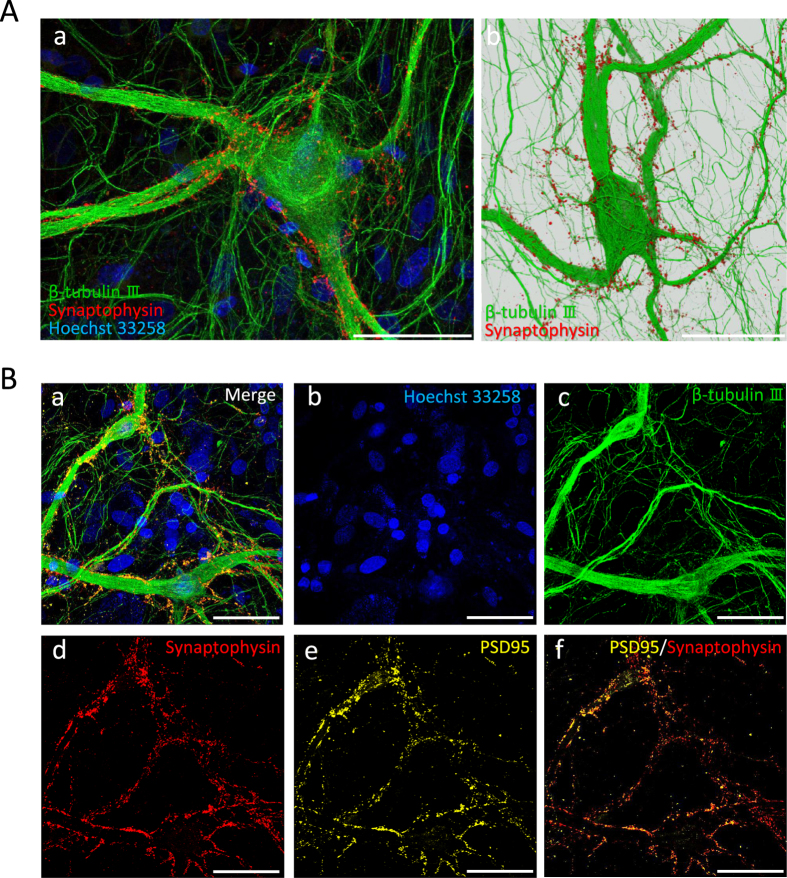
Pyramidal neuron-like morphology and synaptogenesis at 300 DIV. (**A**) Fluorescent image of 300 DIV neuron with pyramidal-shape soma. Scale bars = 50 μm. (a) These 300 DIV neurons have thick primary dendrites. Green: neuronal marker β-tubulin III. Red: presynaptic marker (synaptophysin). Blue: nuclear marker (Hoechst 33258) revealing underlying astrocytes. Synapses were formed around the soma and thick dendrites. (b) 3-dimentional (3D) reconstructed image of Z-axis images acquired using confocal microscopy (see 3D Supplementary Movie 1). (**B**) Formation of pre- and postsynaptic structures at 300 DIV. (a) Merged image showing the formation of synapses among cultured hiPSC-derived cortical neurons. Blue: nuclear marker Hoechst 33258 revealing underlying astrocytes. Green: neuronal marker β-tubulin III. Red: presynaptic marker synaptophysin. Yellow: postsynaptic marker PSD-95. (b) Image showing the nuclei using Hoechst 33258 staining (blue). (c) Image showing neurite formation using β-tubulin III immunostaining (green). (d) Image showing presynapse formation using synaptophysin immunostaining (red). (e) Image showing postsynapse formation using PSD-95 immunostaining (yellow). (f) Merged image showing overlap or close apposition of presynaptic (synaptophysin-positive) and postsynaptic (PSD-95-positive) terminals.

To study progressive changes in the morphological, electrophysiological, and pharmacological properties of human iPS-derived neurons and neural networks, we established a hiPSC–astrocyte co-culture system able to survive for over 1 year. Figure 1A shows a comparison of neuronal size after 112 and 300 days *in vitro* (DIV) as revealed by immunostaining using the neuronal marker β-tubulin III. The neuron at 300 DIV had a larger soma and thicker primary dendrites. The mean (±S.E.) somal area calculated from such images increased from 208 ± 11.9 μm^2^ at 112 DIV to 557 ± 28.8 μm^2^ at 300 DIV (Fig. 1B), and the width of primary dendrites increased from 2.96 ± 0.44 μm at 120 DIV to 7.86 ± 0.59 μm at 300 DIV (n > 100, P < 0.001; Fig. 1C). In addition, neurons acquired a pyramidal-like morphology with apical and basal dendrites (Fig. 2A–a,b) similar in appearance to cerebral cortical neurons *in vivo*. Somal thickness at 300 DIV was approximately 6.4 μm as revealed by Z-axis stacks of confocal images (Supplementary Movie 1). To confirm synaptogenesis at 300 DIV (i.e., the formation of neural networks), we performed immunochemical staining for the presynaptic marker synaptophysin and the postsynaptic marker PSD-95. Synapses were mainly formed around thick dendrites and the soma (Fig. 2A,B).

### Development of spontaneous firing in long-term culture of hiPSC-derived cortical neurons

**Figure 3:**
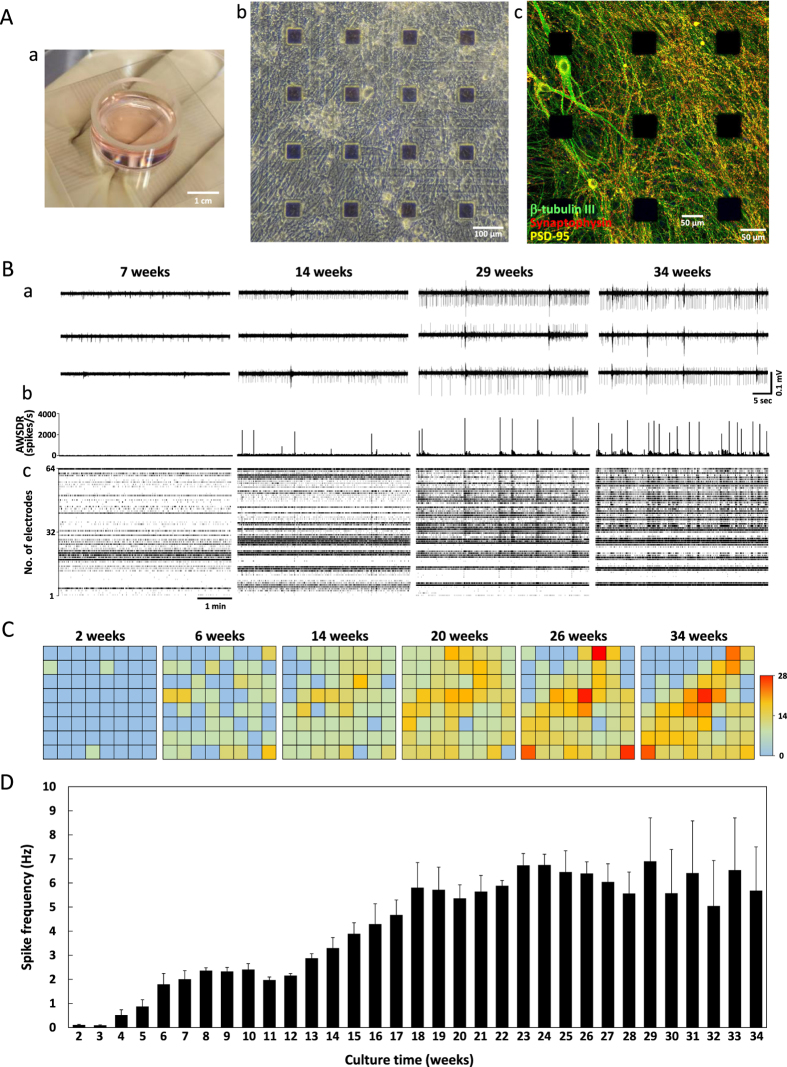
Development of spontaneous firing during a long-term culture of hiPSC-derived cortical neurons. (**A**) hiPSC-derived cortical neurons cultured on an MEA chip. (a) Overview of a MEA. (b) Phase contrast image of hiPSC-derived neurons on a MEA chip at 294 DIV. (c) Immunofluorescent image of neurons on a MEA chip at 300 DIV. Images show the soma and neurites using β-tubulin III (green), presynapse formation using synaptophysin (red), and postsynapse formation using PSD-95 (yellow) immunostaining. (**B**) Changes in spontaneous firing pattern in the same long-term culture at 7, 14, 29, and 34 weeks *in vitro* (WIV). (a) Typical spontaneous firing patterns. (b) Rasters of the array-wide spike detection rate (AWSDR, spikes/s). Bin size is 1 ms. (c) Corresponding raster plots for all 64 electrodes over 5 min. (**C**) Electrode grids colored-coded to indicate mean spontaneous firing frequency from same culture at 2, 6, 14, 20, 26, and 34 WIV. Red indicates electrodes with higher firing frequencies. Scale bar in Hz (maximum: 28 Hz). (**D**) Time course of the average firing frequency per channel from 2 to 34 WIV. Firing frequency (± standard deviation) was calculated as the average of all 64 electrodes from each of the three MEA dishes.

**Figure 4:**
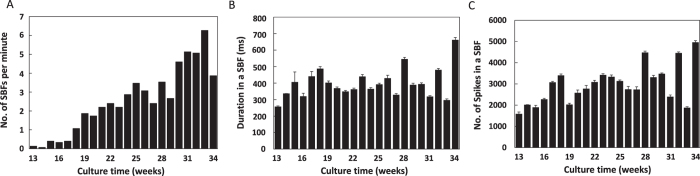
Development of spontaneous synchronized burst firings (SBFs) during long-term culture. (**A**) The number of SBFs per minute (average for 15 min) from 13 to 34 WIV. (**B**) Change in SBF duration over the same period *in vitro* (n = number of SBFs for each weekly 15-min measurement period). (**C**) Change in total number of spikes in SBFs (total number in each weekly 15-min measurement period).

To investigate functional maturation over time in culture, we established co-cultures of hiPSC-derived cortical neurons and astrocytes on MEA chips (Fig. 3A–a) and recorded spontaneous and evoked extracellular field activity once every week. Figure 3A–b is a phase contrast image of neurons at 294 DIV and 3A-c an immunofluorescent image of neurons at 300 DIV growing on the MEA. Human iPSC-derived cortical neurons grown on MEA chips survived over the long-term without cell aggregation, enabling the measurement of distributed network field activity. During long-term culture, neurites and synapses progressively formed to create a dense neuropil among the somata (Fig. 3A–c). Representative examples of spontaneous firing patterns at 7, 14, 29, and 34 weeks *in vitro* (WIV) revealed increased activity with culture time, particularly from 14 to 29 WIV. Figure 3B–b,c show the array-wide spike detection rate (AWSDR) raster plots and individual raster plots for all 64 electrodes, demonstrating the increase in the number of spikes and field spike amplitude with increased WIV. Moreover, the temporal correspondence with spikes in multiple electrode channels indicates that these AWSDR events correspond to synchronized burst firings (SBFs). Figure 3C shows the time course of the change in spontaneous firing rate for the individual electrodes within the 8 × 8 grid from 2 to 34 WIV and Fig. 4D the average of three MEA cultures. At 2 WIV, spontaneous firing was detected at only three electrodes, whereas at 20 WIV, spontaneous firing was detected at 62 electrodes. Although the number of spike-detecting electrodes decreased from 27 to 34 WIV because of cell migration, there was a substantial increase in the number of electrodes detecting high-frequency spikes over 10 Hz (16 and 21 channels, respectively) and over 20 Hz (4 and 8 electrodes, respectively) (maximum = 27.6 Hz). Figure 3D shows the time course of the change in the average firing frequency up to 34 weeks (n = 3 MEA dishes) calculated by dividing the total number of spikes by the total number of electrodes. Firing frequency (average per electrode) increased to about 2 Hz by 6 weeks, remained relatively constant up to 12 weeks and then rose again to a plateau of approximately 5 Hz from 18 to 34 weeks (Fig. 3D).

A critical characteristic of functional cortical networks *in vivo* is the development of spontaneous SBFs as these events mediate synaptic signal propagation. Occasional SBFs were observed at 10–13 weeks from most electrodes but there was a substantial increase in frequency by 29–34 WIV (Fig. 3B, Fig. 4A,C). In contrast to this progressive increase in SBF frequency, mean spikes per burst and burst duration reached a plateau relatively early (by about 18 weeks). Moreover, there was a rough correspondence between the mean number of spikes per SBF and SBF duration, suggesting a relatively constant firing frequency within bursts. These results indicate that hiPSC-derived cortical neurons eventually form functional networks that are able to generate SBFs during long-term culture.

### Pharmacological properties of spontaneous firing

**Figure 5:**
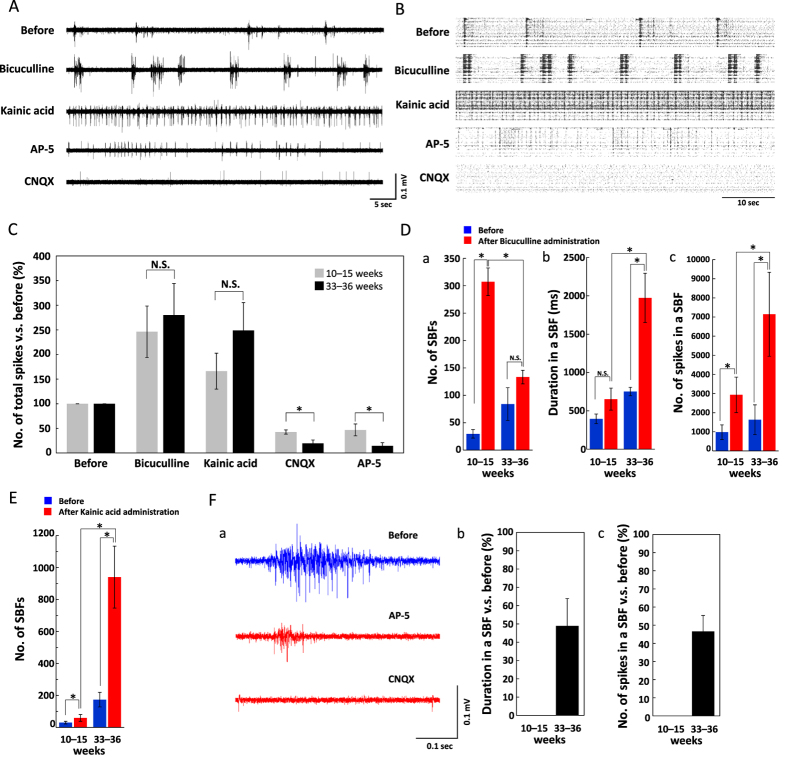
Pharmacological properties of spontaneous firing activity. (**A**) Typical spontaneous firing at the same electrode in 33–36 WIV cultures before (top) and after the administration of the indicated receptor antagonist or agonists (10 μM bicuculline, 5 μM kainic acid, 50 μM AP-5, 50 μM CNQX). (**B**) Raster plots of spontaneous firing for 1 min from all 64 electrodes before and after drug administration. (**C**) Total number of spikes from all 64 electrodes before (100%, baseline) and after drug administration at 10–15 and 33–36 WIV. Comparisons between 10–15 (gray) and 33–36 WIV (black) were obtained using the same cultures. Although absolute numbers of spikes at 10–15 and 33–36 WIV were different, the proportional changes did not differ between ages for bicuculline and kainic acid. In contrast, the decrease in total spikes induced by CNQX and NMDA administration differed by WIV, with greater proportional effects of both agents at 33–36 WIV. (n = 3 MEA dishes, *p < 0.05). (**D**) Changes in synchronized burst firing (SBF) due to bicuculline administration at 10–15 and 33–36 WIV. (n = 3 MEA dishes, *p < 0.05). (a) Number of SBFs over the 30 min before (blue) and after (red) bicuculline administration at 10–15 and 33–36 WIV. (b) SFB duration and (c) number of spikes per SBF at 10–15 and 33–36 WIV before and after bicuculline administration, *p < 0.05). (**E**) Number of SBFs in the 30 min before and after 5 μM kainic acid administration at 10–15 and 33–36 WIV (n = 3 MEA dishes, *p < 0.05). (**F**) Change in SBFs following AP-5 and CNQX administration at 10–15 and 33–36 WIV. (a) Typical SBF waveforms before (blue) and after the administration of AP-5 (red) or CNQX (red) at 33–36 WIV. SBFs disappeared after CNQX administration. SBFs were also completely abolished by AP-5 at 10–15 WIV but were only shorter at 33–36 WIV. (b) Change in SBF duration and (c) number of spikes per SBF following AP-5 administration at 10–15 and 33–36 WIV.

To evaluate the maturation of synaptic function with time in culture, we measured changes in spontaneous firing following application of the GABA_A_ antagonist bicuculline (10 μM), the KA subtype glutamate receptor (GluR) agonist kainic acid (KA, 5 μM), the AMPA/kainate GluR antagonist CNQX (50 μM), or the NMDA GluR antagonist AP-5 (50 μM) at 10–15 WIV and 33–36 WIV (Fig. 5A,B). Bicuculline significantly increased the firing rate, by 246% ± 52.8% at 10–15 WIV and 280% ± 64.1% at 33–36 WIV, relative to baseline (Fig. 5C). Although the magnitude of the increase in mean spike frequency did not differ between culture ages, the mean increase in SBF number over 30 min post-administration was markedly higher in the 10–15 WIV cultures (29.7 ± 7.7 to 307.3 ± 25) compared with that in 33–36 WIV cultures (84.0 ± 29.8 to 133.3 ± 12.4) (Fig. 5D–a). In contrast, both the baseline and post-bicuculline mean SBF durations were significantly longer at 33–36 WIV (752.3 ± 55.1 ms to 1973.0 ± 320.2 ms) compared with those at 10–15 WIV (395.7 ± 63.1 to 653.1 ± 143.9) (Fig. 5D–b), with a concordant difference in the total mean number of total spikes over 30 min (10–15 WIV: 980.6 ± 380.3 to 2930.7 ± 928.2; 33–36 WIV: 1631.9 ± 777.9 to 7144.5 ± 2193.1) (Fig. 5D–c). Thus, more functionally developed cultures exhibited lower bicuculline-induced SBF frequency but longer individual SBFs.

Kainic acid administration increased the firing rate by 166% ± 36.5% at 10–15 WIV and 249% ± 56.6% at 33–36 WIV (Fig. 5C). Again, there was no significant difference in the magnitude of the increase between culture ages, but both the baseline and KA-induced increase in total SBF number were markedly higher at 33–36 WIV (173.7 ± 46.0 to 940.3 ± 193.5) compared with those at 10–15 WIV (30.0 ± 8.0 to 59.3 ± 21.4) (Fig. 5E), with no age-dependent difference in mean total spike number (Fig. 5C) or SBF duration (not shown). Thus, 33–36 WIV cultures were more sensitive to KA-induced hyperexcitability than 10–15 week WIV cultures.

In 10–15 WIV cultures, CNQX decreased the firing rate to 42.6% ± 12.1% and AP-5 to 46.9% ± 4.28% of baseline. In 33–36 WIV cultures, these same concentrations evoked larger magnitude decreases in firing rates, to 19.5% ± 6.69% and 14.4% ± 6.65%, respectively (Fig. 5C). No SBFs were observed following CNQX administration at either age, but SBFs were observed in 33–36 WIV cultures after AP-5 administration (Fig. 5B). Figure 5F–a shows typical changes in burst firing pattern at an electrode before and after CNQX or AP-5 administration. At 33–36 WIV, AP-5 administration decreased SBF duration to 48.9% ± 14.9% and number of spikes to 46.6% ± 8.8% of baseline (Fig. 5F–b,c), while all SBFs were eliminated by CNQX. These results indicate that both AMPA and NMDA receptors contribute to SBFs; however, only AMPA receptor activation is necessary for SBF generation in hiPSC-derived cortical networks, while NMDA receptor activity modulates spontaneous SBF duration. In sum, these results indicate that both AMPA and NMDA receptors more efficiently drive neuronal spiking at 33–36 WIV compared with that at 10–15 WIV. Overall, spontaneous activity in 33–36 WIV cultures was more sensitive to GABA and glutamate receptor modulators, indicative of greater functional maturation.

### Pharmacology of evoked responses

**Figure 6:**
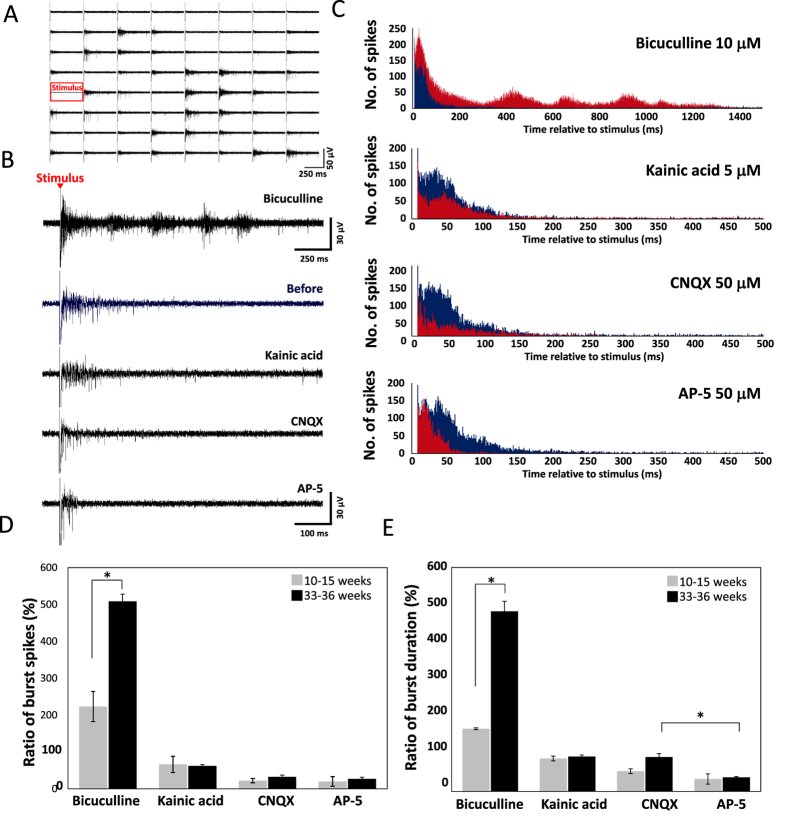
Pharmacological properties of evoked responses. (**A**) Typical evoked responses from each of 64 electrodes following a single test stimulus at 33–36 WIV. Red square shows stimulus site (electrode 33). (**B**) Typical evoked responses before (top) and after the administration of the indicated neurotransmitter receptor agonist or antagonist at 33–36 WIV. Red triangle shows stimulus time and stimulus artifacts. (**C**) Post-stimulus time histogram (PSTH) (Sum of 10 individual responses at 64 electrodes, bin size = 1 ms) at 33–36 WIV. Blue and red indicate before and after drug administration, respectively. (**D**) Ratio of the number of evoked spikes after versus before drug administration at 10–15 WIV (gray) and 33–36 WIV (black) (n = 3 MEA dishes, *p < 0.05). (**E**) The ratio of evoked burst duration after versus before drug administration. (n = 3 MEA dishes, *p < 0.05).

The effects of these same drugs were then examined on evoked responses. Test stimuli were applied 10 times at 30-s intervals before and after drug administration. Figure 6A shows typical evoked responses from 64 channels following stimulation by electrode 33 in a 33–36 WIV culture. Evoked burst firings were observed from over one-third of the electrodes. We then compared evoked responses between 10–15 and 33–36 WIV cultures before and after bicuculline (10 μM), KA (5 μM), CNQX (50 μM), or AP-5 (50 μM) administration. Individual pharmacological experiments were performed at 2-day intervals to minimize residual drug effects. Typical SBF responses in a 33–36 WIV culture are shown in Fig. 6B and post-stimulus time histograms (PSTHs) for all 64 electrodes over 10 individual post-stimulus epochs before (blue) and after (red) drug administration in Fig. 6C. Application of 10 μM bicuculline increased both burst duration and the total number of spikes. These augmented bursts consisted of a primary early peak with substantially more spikes than observed before bicuculline administration as well as multiple secondary peaks of elevated spike frequency not observed in the absence of bicuculline. Bicuculline increased the number of spikes by 223% ± 40.8% at 10–15 WIV and by 508% ± 19.1% at 33–36 WIV relative to baseline (Fig. 6D), and burst duration by 168% ± 2.41% at 10–15 WIV and 482% ± 26.9% at 33–36 WIV (Fig. 6E). In contrast, 5 μM KA decreased the number of evoked spikes at both ages, to 66% ± 22% of baseline at 10–15 WIV and to 62% ± 3.7% of baseline at 33–36 WIV (Fig. 6D), and burst duration to 88% ± 6.3% of baseline at 10–15 WIV and 93% ± 4.3% of baseline at 33–36 WIV (Fig. 6E), likely because the increase in spontaneous activity (Fig. 5C,E) induced frequent spike refractory periods. The application of 50 μM CNQX significantly decreased the number of spikes at both ages but not burst duration at 33–36 WIV (Fig. 6C). The number of spikes was reduced to 22% ± 5.8% of baseline at 10–15 WIV and to 32% ± 4.9% at 33–36 WIV, whereas burst duration was 54% ± 6.5% of baseline at 10–15 WIV and 92% ± 9.1% at 33–36 WIV. Alternatively, AP-5 application decreased both the number of spikes and burst duration (Fig. 6C) regardless of WIV. The number of spikes was reduced to 20% ± 13% of baseline at 10–15 WIV and to 27% ± 4.6% of baseline at 33–36 WIV, and burst duration to 33% ± 13% and 38% ± 1.7% at 10–15 and 33–36 WIV, respectively. It should be noted that the number of spikes within 50 ms post-stimulus was actually larger following AP-5 application (Fig. 6C). Administration of AP-5 also reduced burst spike number and duration, and in this case the magnitude was significantly larger than for CNQX at 33–36 WIV (Fig. 6E, P < 0.02). From these experiments, we confirm that both AMPA and NMDA receptors are active during evoked responses, with NMDA receptor activity having a greater influence on burst duration and AMPA receptor activity having a greater influence on early post-stimulus spike frequency.

### Induction of epileptiform activity and effects of anti-epilepsy drugs (AEDs)

To evaluate the utility of these human iPS-derived neural networks for modeling human disease states and drug screening, we examined chemically evoked epileptiform activity. Electrophysiological seizes were induced by pentylentetrazole (PTZ), the most widely used chemical convulsant in animal models to screen for new anti-epilepsy drugs (AEDs). We also examined the anti-convulsant effects of two common clinical AEDs, phenytoin and sodium valproate (VPA). Phenytoin experiments were conducted at 13 weeks (89 DIV) and 20 weeks (139 DIV), and VPA experiments at 15 weeks (99 DIV) and 46 weeks (317 DIV) using the same cultures.

**Figure 7:**
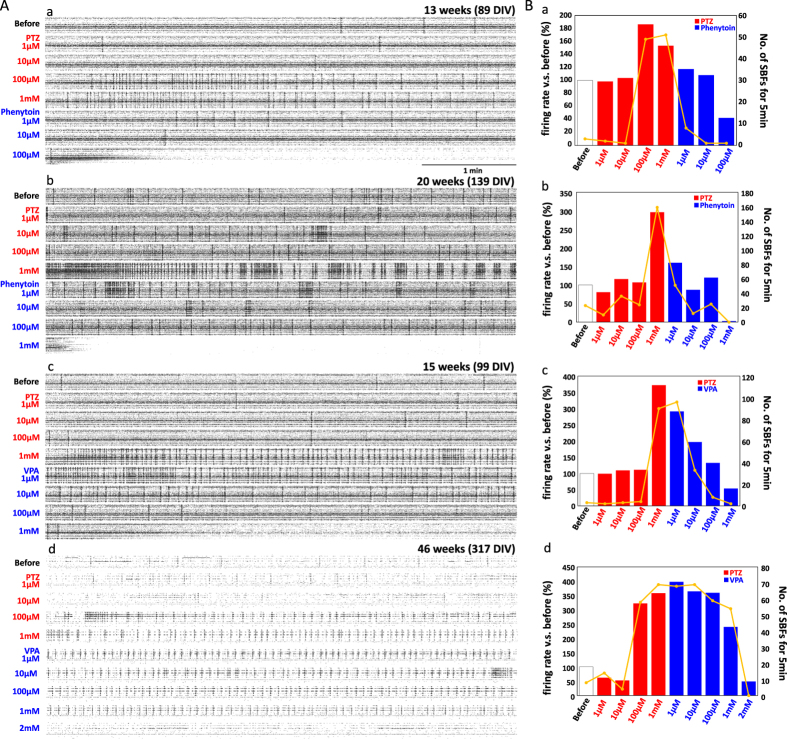
Induction of epileptiform activity and anticonvulsant effects of anti-epilepsy drugs (AEDs). (**A**) Induction of epileptiform activity using pentylentetrazole (PTZ) and the suppressive effect of phenytoin. PTZ was added to the culture medium at increasing concentrations (1 μM, 10 μM, 100 μM, and 1 mM). Phenytoin was then added (1 μM, 10 μM, 100 μM, and 1 mM). Effects on network activity are clearly revealed by the raster plots (64 channels for 5 min). The appearance of black vertical lines indicates SBFs. (a) The raster plots at 13 WIV (89 DIV). (b) Changes in firing rate versus before (%) and number of SBFs during drug treatment at 13 WIV. Red and blue bars show the firing rates at each PTZ and phenytoin concentration, respectively. Yellow polygonal line shows the number of SBFs over each 5-min application of PTZ or phenytoin at the indicated concentration. (c) The raster plots at 20 WIV (139 DIV). (d) Changes in firing rate and number of SBFs at 20 WIV. (**B**) Effect of sodium valproate (VPA) (1 μM, 10 μM, 100 μM, 1 mM, and 2 mM). (**a**) The raster plots at 15 WIV (99 DIV). (b) Changes in firing rate and number of SBFs at 15 WIV. (c) The raster plots at 46 WIV (317 DIV) t. (**d**) Changes in firing rate and number of SBFs at 46 WIV.

Pentylentetrazole induced a rapid increase in SBFs at 100 μM and 1 mM in all experiments. In the experiment on a 13 WIV culture shown in Fig. 7A–a,B–b, SBF number increased from 3 in the 5 min before PTZ administration to 49 in the first 5 min after addition of 100 μM PTZ and to 51 in the 5 min after addition of 1 mM PTZ. Total firing rate also increased, to ~150% of baseline. After the sequential administration of 1 and 10 μM phenytoin, SBF number was reduced to 8 and 1, respectively, and total firing rate to 117 and 108% of pre-PTZ baseline, respectively. The administration of 100 μM phenytoin reduced firing frequency dramatically compared with baseline (Fig. 7A–a,B–b). At 20 weeks, high frequency SBFs (over 1 Hz) occurred immediately after 1 mM PTZ administration (Fig. 7A–b,B–b). The number of SBFs increased to 296% of baseline (to 159 bursts) during the first 5 min post-PTZ (Fig. 7B–b), whereas 1 μM phenytoin reduced the total number to 159% of baseline (51 bursts) and 10 μM administration to 86% (21 bursts). High frequency SBFs disappeared in 100 μM phenytoin. Unlike at 13 WIV, however, neither spontaneous firing rate nor SBFs disappeared in the presence of 100 μM phenytoin. Such suppression required 1 mM. These dose-dependent changes in firing over time are shown in Supplementary Movie 2. At both 13 and 20 WIV, the induction of epileptiform activity was induced by PTZ administration and suppressed by phenytoin, but this AED appeared more potent in younger cultures.

Valproic acid also suppressed PTZ-induced epileptiform activity. In the 15 WIV culture shown in Fig. 7A–c,B–c, SFB number rose from 3 to 90 and firing rate to 371% of baseline after the administration of 1 mM PTZ. Although 1 μM VPA was ineffective in reducing SFBs, SFB number was gradually reduced to 33 in 10 μM and to 8 in 100 μM VPA. All three doses reduced the firing rate, to 291% at 1 μM, 197% at 10 μM, and 133% of pre-PTZ baseline at 100 μM VPA, whereas 1 mM completely suppressed SBFs within 20 s and reduced firing rate below baseline (Fig. 7A–c,B–c). At 46 weeks, 100 μM PTZ increased SFB number from 8 to 58 and firing rate to 322% of baseline, whereas 1 mM PTZ increased SFB number to 69 and firing rate to 358% of baseline (Fig.7A–d,B–d). Valproic acid at 1, 10, and 100 μM did not significantly reduce the number of SBFs or firing rate (Fig. 7A–d,B–d). In fact, 1 mM VPA reduced SBF number and firing rate only slightly. At 2 mM, VPA completely suppressed SBFs and reduced the total spike frequency to near baseline (Fig. 7A–d,B–d). Although both phenytoin and VPA have anticonvulsant efficacy in this culture system, phenytoin was more potent. Moreover, the efficacy of VPA was lower in older cultures.

**Figure 8:**
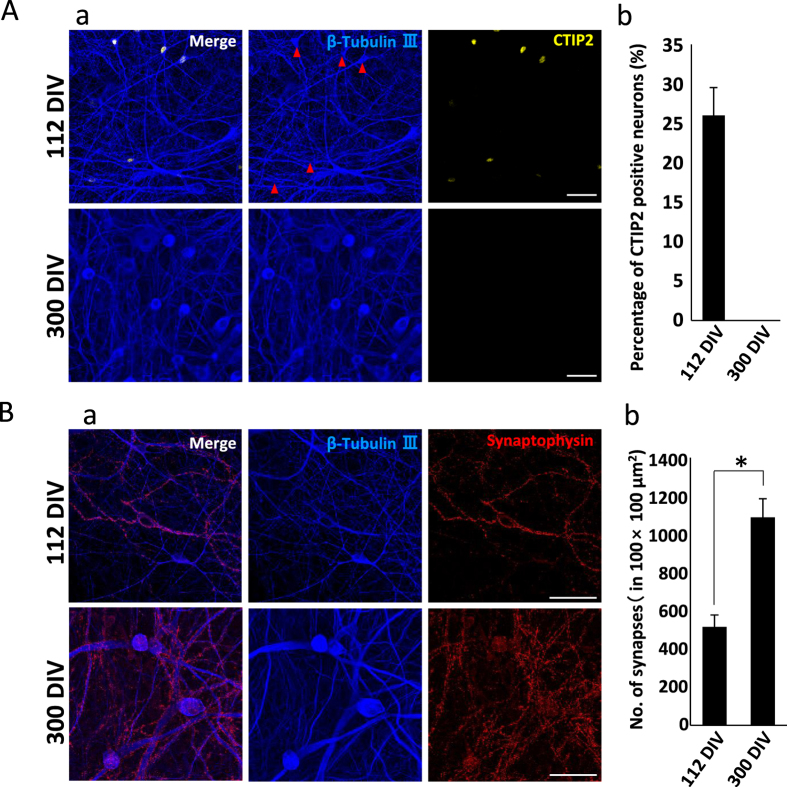
Comparison of immature neuron number and synaptic density between 112 and 300 DIV cultures. (**A**) Immunofluorescent staining for CTIP2 at 112 and 300 DIV. (a) Fluorescent images of CTIP2 staining for the specific labeling of immature cortical neurons (yellow) and β-tubulin III (blue) for all neurons. Upper and lower images show hiPSC-derived cortical neurons at 112 and 300 DIV, respectively. Red circles indicate CTIP2-positive neurons. Scale bars = 50 μm. (b) Percentage of immature (CTIP2-positive) neurons at 112 and 300 DIV. (n = 22) (**B**) Immunofluorescent analysis of synapse density at 112 and 300 DIV. (a) Fluorescent images of synaptophysin for the specific labeling of presynaptic terminals (red) and β-tubulin III (blue) for neurons. Upper and lower images show hiPSC-derived cortical neurons at 112 and 300 DIV, respectively. Scale bars = 50 μm. (b) The number of synapses in the 100 × 100 μm^2^ area around each soma at 112 DIV (515 ± 62.6) and 300 DIV (1088 ± 98.3) (n = 15 synaptophysin-positive neurons, *P < 0.01).

In aggregate, these results indicate that individual neurons mature and accumulate substantially greater numbers of synaptic contacts from ~13–15 to ~27–40 WIV. To confirm this, we stained 112 and 300 DIV cultures with a marker for immature neurons (CTIP2) and calculated mean synaptic density. Indeed, the young cultures demonstrated numerous immature neurons, whereas none were detected in 300 DIV cultures (Fig. 8A). Further, the older cultures exhibited significantly higher synaptic density (Fig. 8B).

## Discussion

We succeeded in maintaining hiPSC-derived cortical neurons in culture for more than one year and detected the progressive development of spontaneous and evoked spiking, typical responses to neurotransmitter receptor modulators, and epileptiform bursting by suppression with clinical anticonvulsants. Cultured hiPSC-derived cortical neurons at 300 DIV were morphologically mature, exhibiting thick dendrites, a large soma, numerous somal and dendritic synapses, and an extensive surrounding neuropil (Figs 1 and 2, and supplementary movie 1) resembling cortical pyramidal neurons *in vivo*. We also confirmed progressive synaptogenesis by the co-localization of pre- and postsynaptic markers around soma and thick dendrites (Fig. 2). Moreover, this progressive synaptogenesis paralleled the developmental increases in spontaneous and evoked spiking and responsivity to GABAergic and GluR antagonists. On the other hand, synapses were not formed around many thin neurites (Fig. 2A), some of which are likely axons, indicating that only functional synapses remained after synaptic pruning^26^.

Spontaneous activity evolved over 34 weeks, increasing from less than 1 Hz at 2 WIV to over 5 Hz at 18 WIV, underscoring the necessity of long-term culture for the formation of functional networks. Some electrodes even recorded spike frequencies over 20 Hz at 25 WIV or older. Spike patterns consisted of both tonic spike trains and SBFs (e.g., Fig. 3B), which are electrophysiological hallmarks of GABAergic and glutamatergic neurons, respectively. Thus, these hiPSC-derived cortical neuronal networks likely contained GABAergic interneurons as well as (more numerous) glutamatergic pyramidal neurons.

Synchronized spike bursts are critical for information transfer within the cortex and are indicators of functional maturation. Such SBFs were first observed at 10–13 WIV and increased in frequency up to approximately 30 WIV. In rat cortical neurons at 1/4 cell density, synchronized firing attributable to chemical synaptic transmission occurred after only 1 week in culture^24^, suggesting that hiPSC-derived cortical neurons require much longer to achieve functional maturation *in vitro*. Nonetheless, this extra time is reasonable considering the advantages for translational research presented by human neurons.

We were also able to record spontaneous activity for over 77 weeks (544 days) (supplementary Fig. S1). In this experiment, spike frequency increased up to approximately 25 WIV, and although it decreased after 30 WIV, it was maintained at approximately 3 Hz at 72 weeks. We also confirmed that SBFs were generated in cultures at 72 WIV (Fig. S1-C). These long-term recordings revealed an additional unusual phenomena, the presence of “super SBFs” longer than 5 s at both the network (Fig. S1-D) and single neuron level (Figs S1-E and C-e). These results indicate that hiPSC-derived cortical neurons over 1 year *in vitro* acquire the potential for maintained burst firing. In rat cortical neurons *in vitro*, the prolongation of SBFs was also reported with DIV^24^,^27^,^28^, but the time scale of the change is over ten times longer in hiPSC-derived cortical neurons. The future of SBFs in rat or mouse iPSC-derived cortical neurons was not reported. We suggest that the time scale of these spontaneous burst episodes is a seminal difference between rat and human neurons. However, the frequency and rhythm of SBFs differed within each measurement period, so comparative analyses of SBFs remain a challenge.

These spontaneous and evoked responses also showed progressive changes in pharmacological response. The relative increase in spike rate following bicuculline administration did not differ between 10–15 and 33–36 WIV, whereas the increase in SBF was significantly smaller at 33–36 WIV. On the other hand, SBF duration and spikes per SBF were higher at 33–36 WIV compared with those at 10–15 WIV. We suggest that these increases in SBF duration and spikes per burst are due to recurrent excitatory inputs present in mature networks and that the decrease in SBF number results from refractory periods created by these longer duration SBFs. In fact, the repetitive peaks in spike frequency observed after the primary burst (Fig. 6C) suggest such recurrent excitation. In addition, we found a reciprocal relationship between SBF duration and number (Fig. S2), consistent with an increase in refractory period length or frequency at higher burst frequencies.

Predictable and stable responses are important for the standardization of drug screening using the MEA system. Kainic acid, which is known to cause convulsions in cerebral cortex slices *in vitro*^29^,^30^,^31^,^32^, evoked a large increase in spontaneous SBF number, particularly at 33–36 WIV. Thus, these networks mirrored the response of cortical slices. The suppressive effects of CNQX and AP-5 on spike number were also larger at 33–36 WIV compared with those at 10–15 WIV (Fig. 5C). These results suggest that hiPSC-derived cerebral neurons at 33–36 weeks express mature glutamatergic receptors with full complements of KA, AMPA, and NMDA receptors. In particular, the detection of fully functional NMDA responses is important because the NMDA receptor mediates several forms of neuroplasticity related to learning^33^,^34^. Moreover, NMDA overactivity is a key pathogenic event in numerous acute and chronic neurodegenerative diseases.

These receptor modulators had largely analogous effects on evoked responses. Bicuculline also increased evoked SBF duration and spike number, and cultures were more sensitive to bicuculline-evoked hyperactivity at 33–36 WIV than at 10–15 WIV, presumable because of the contribution from recurrent inputs. AP-5 administration at 33–36 weeks markedly reduced SFB duration (Fig. 6C), suggesting that these kinetically slower GluRs are major contributors to recurrent activity. In contrast, the AMPA antagonist CNQX reduced the initial evoked spike burst but not the duration, indicating that the AMPA and NMDA receptors plays an important role in early and late phase of SBF, respectively. In sum, both spontaneous firing and evoked responses were modulated by GABAergic and glutamatergic receptor modulators, although the effective concentrations (ECs) remain to be determined.

To investigate the causes of these age-dependent differences in electrophysiological and pharmacological properties, we compared the numbers of immature neurons (using the marker CTIP2^35^) and synaptic densities between 112 and 300 DIV cultures. At 112 DIV, 26.0% ± 3.52% of neurons were still CTIP2-positive and the synaptic density was about 50% lower than at 300 DIV (Fig. 8B–b). In addition, some neurons at 112 DIV had not yet formed synapses (Fig. 8B–a). These results confirm that hiPSC-derived cortical neuronal networks at 112 DIV are not fully mature and that longer-term culture is necessary for maturation.

We also observed the induction of epileptiform activity by PTZ and suppressive effects by clinical AEDs (phenytoin and VPA). Phenytoin depressed SBF and firing rate at 1 μM. Phenytoin is a use-dependent blocker of voltage-gated sodium channels that inhibits rapid burst firing, while allowing lower-frequency non-burst firing^36^. In rat neocortical neurons, phenytoin diminished sodium currents at concentrations as low as 1 μM^37^,^38^. Similarly, low micromolar phenytoin concentrations preferentially blocked SBFs over single spikes in human iPSC-derived neuronal networks. The anticonvulsant effect of VPA is generally ascribed to the enhancement of GABAergic inhibitory neurotransmission^39^,^40^. VPA administration dose-dependently decreased firing rate and the number of SBFs. However, at 46 WIV (317 DIV), suppression was not achieved even at 1 mM, whereas 2 mM VPA completely suppressed all activity. At higher concentrations, VPA also altered the kinetics of the sodium current in cortical neurons from human patient by shifting the voltage-dependence of steady-state inactivation to more hyperpolarized potentials^41^. The suppressive effects at 46 WIV may therefore be due to changes in sodium current kinetics. Another possibility is that GABAergic neurons or GABAergic synapse density was decreased at 46 WIV compared with that at 15 WIV. Indeed, the reduced tonic firing at 46 WIV compared with that at 15 WIV (Fig. 7B) is suggestive of reduced GABAergic neuron number. Future studies using GABAergic markers are needed to determine the relative proportions of GABAergic and glutamatergic neurons during culture maturation.

In addition to these differences in AED responses, we also found differences in PTZ potency with time *in vitro*. The SBF and total firing rates were higher at 20 WIV than at 13, 15, or 46 WIV. In addition, SBFs showed greater regularity at 46 WIV compared with that at 13, 15, and 20 WIV. Although the age-dependent mechanisms are unknown, these result indicate that time in culture is an important factor for the evaluation of AEDs using PTZ and thus requires standardization.

Although PTZ is the most widely used proconvulsant for testing new AEDs, seizures induced by PTZ in animals are not responsive to all potential AEDs. For instance, phenytoin is not effective against PTZ in rats or mice^42^. Given the responsivity demonstrate here, we suggest that the human iPSC-derived neuron culture is a useful model system to investigate the effects of common AEDs and an alternative to animal experiments for drug screening. In addition, our assay can be adapted to iPSC-derived neurons from epilepsy patients.

In conclusion, we examined the electrophysiological and pharmacological properties of cultured hiPSC-derived cortical neuronal networks and found that functional maturation requires at least 20–30 weeks. Nonetheless, long-term culture of hiPSC-derived neuronal neurons on MEAs proved useful for neuropharmacological and neurotoxicological assays. Our results also provide an important indication for the international standardization of evaluation procedures using *in vitro* human neurons.

## Materials and Methods

### Culture of hiPSC-derived cerebral cortical neurons

Human induced PSC-derived cerebral cortical neurons (hyCCNs; ax0026F, Axol Bioscience Inc., UK)^43^ were cultured at 1.0 × 10^6^ cells/cm^2^ on 64-channel MEA chips (MED-P515A; Alpha Med Scientific) coated with Axol Sure Bond Coating Solution (Axol Sure Bond ^TM^ Coating solusion; ax0041, Axol Bioscience Inc.) at 37 °C in a 5% CO_2_/95% air atmosphere. For culture on MEAs, a φ3.4-mm glass ring was placed in the middle of the MEA probe at the location of the electrode array, and 100 μL of cell suspension (1.0 × 10^6 ^cells/mL) was seeded inside the ring. After 1 h, culture medium Neural Maintenance Media kit (ax0031a&b, Axol Bioscience Inc., UK) supplemented with 100 U/mL penicillin/streptomycin (168-23191, Wako) and 20% conditioned media (described below) was applied around the ring, and the ring carefully removed. Conditioned medium was obtained from the culture supernatant of rat primary cortical astrocytes in neurobasal medium supplemented with 2% v/v B27, 10% v/v fetal bovine serum, 100 U/mL penicillin/streptomycin (all from Invitrogen), and 0.074 mg/mL l-glutamine (21051-24, GIBCO). Next, rat astrocytes were seeded at 1 × 10^4^ cells per MEA chip and the co-cultures maintained at 37 °C in a 5% CO_2_/95% air atmosphere. Half of the media was exchanged every 5 to 7 days.

### Immunocytochemistry

Sample cultures were fixed with 4% paraformaldehyde in phosphate-buffered saline (PBS) on ice (4 °C) for 10 min, followed by methanol on ice (−20 °C) for 10 min. Fixed cells were incubated with 0.2% Triton X-100 in PBS for 5 min, followed by preblock buffer (0.05% Triton-X and 5% goat serum in PBS) at 4 °C for 1 h, and finally with preblock buffer containing a specific primary antibody (1:1000) at 4 °C for 24 h. The primary antibodies used were mouse anti-β-tubulin III (T8578, Sigma–Aldrich), rabbit anti-synaptophysin (MAB329, MILLIPORE), goat anti-PSD95 (ab12093, Abcam) and rat anti-CTIP2 (ab18465, Abcam) for the specific labeling of neurons, presynaptic terminals, postsynaptic junctions, and immature cortical neurons, respectively. Immunolabeling was visualized by incubation in an appropriate secondary antibody (anti-mouse 488 Alexa Fluor (ab150109, Abcam), anti-rabbit 546 Alexa Fluor (A11010, Lifetechnologies), anti-rat 647 Alexa Fluor (ab150155, Abcam), or anti-goat 680 Alexa Fluor (ab175776, Abcam), Molecular Probes; 1:1000 in preblock buffer) for 1 h at room temperature. Cell nuclei were counterstained using 1 μg/ml Hoechst 33258 for 1 h at room temperature. Stained cultures were washed twice in preblock buffer (5 min/wash), rinsed twice using PBS, and viewed using a confocal microscope (TCS SP8, Leica). Image intensity was adjusted using ImageJ software (NIH). Soma size and thickness of dendrites were also calculated using ImageJ software.

### Extracellular recording

Spontaneous and evoked extracellular field potentials were acquired at 37 °C under a 5% CO_2_ atmosphere using a 64-channel MEA system (MED64-Basic; Alpha Med Scientific) at a sampling rate of 20 kHz/channel. Signals were low-pass filtered at 100 Hz and stored on a personal computer. Spontaneous firing was recorded every week for up to 72 weeks.

### Pharmacological tests

Spontaneous recordings were obtained for 60 min before treatment and again after the addition of one of the following transmitter receptor agonists and antagonists to the culture medium: the GABA_A_ receptor antagonist bicuculline (10 μM, 026-17611, Wako), kainate receptor agonist kainic acid (5 μM, ab12100, Abcam), AMPA/kainate receptor antagonist 6-cyano-7-nitroquinoxaline-2,3-dione (CNQX, 50 μM; C239-5MG, Sigma–Aldrich), or NMDA receptor antagonist D-(−)-2-amino-5-phosphonopentanoic acid (AP-5, 50 μM; ab120003, Abcam). To evaluate the effects of these compounds on evoked responses, we applied a single bipolar test stimulus (100 μs at + 20 μA, followed by 100 μs at −20 μA) to an electrode every 30 s for 60 min before and after drug administration. The cultures were kept in a CO_2_ incubator between recordings and drug administration. To avoid the residual effects of drug administration and medium changes, each drug experiment was carried out at intervals of two days.

To investigate whether hiPSC-derived cerebral cortical neurons can generate epileptiform activity, we administered 1,5-pentamethylenetetrazole (PTZ, 1 μM to 1 mM; P0046, Tokyo Chemical Industry Co) after various culture durations. Spontaneous firing was recorded for 5 min at each concentration. Then, to evaluate the effects of anti-epilepsy drugs, phenytoin (1 μM, 166-12082, Wako) or sodium valproate (VPA, 2 mM; 197-09722, Wako) was added to the medium.

### Data analyses

Electrophysiological activity was analyzed using Mobius software (Alpha Med Scientific) and MATLAB. A spike was counted when the extracellularly recorded signal exceeded a threshold of ± 5 σ, where σ is the standard deviation of the baseline noise during quiescent periods. To detect and analyze synchronized burst firings (SBFs), the array-wide spike detection rate (AWSDR, spikes/s) from the 64 electrode array was used with a 1 ms bin size as shown in Fig. 3B–b. Firstly, if the inter-spike interval was within 10 ms, these spikes were defined to same SBF. Secondly, if the maximum of AWSDR (bin size, 1 ms) in a SBF is under 5,000 spikes/s or the summation of AWSDR during a SBF is under 20,000 spikes/s, these data sets were eliminated from SBF. Finally, if inter-SBF interval was under 200 ms, we define the same SBF.

To evaluate the changes in evoked responses after drug administration, both pre- and post-drug spikes were measured for 500 ms after the test stimulus or for 1500 ms after bicuculline administration. The initial 6 ms after the test stimulus containing the stimulus artifact was excluded from the analysis. We computed the post-stimulus time histogram (PSTH), where the post-stimulus time window was divided into 1-ms bins.

All data are expressed as mean ± standard error (S.E.); differences between treatment conditions or culture duration were evaluated using two-tailed paired Student’s *t*-test.

## Additional Information

**How to cite this article**: Odawara, A. *et al.* Physiological maturation and drug responses of human induced pluripotent stem cell-derived cortical neuronal networks in long-term culture. *Sci. Rep.* **6**, 26181; doi: 10.1038/srep26181 (2016).

## Supplementary Material

Supplementary Movie 1

Supplementary Movie 2

Supplementary Information
