# Supplementary material for: Physiological maturation and drug responses of human induced pluripotent stem cell-derived cortical neuronal networks in long-term culture
Source: Sci Rep. 2016 May 18;6:26181. doi: 10.1038/srep26181 (PMC4870631; doi:10.1038/srep26181)
Supplement: Supplementary Information [file srep26181-s3.pdf]

Supplementary information

Physiological maturation and drug responses of human induced pluripotent stem cell-derived cortical neuronal networks in long-term culture

A. Odawara[a, b], H. Katoh[a], N. Matsuda[a], I. Suzuki[a*]

[a]Department of Electronics, Graduate School of Engineering, Tohoku Institute of Technology, 35-1 Yagiyama Kasumicho, Taihaku-ku, Sendai, Miyagi, 982-8577, Japan

[b]Japan Society for the Promotion of Science

[*]Corresponding author:

Ikuro Suzuki

Tel: +81-22-305-3219

Fax: +81-22-305-3219

E-mail address: i-suzuki@tohtech.ac.jp

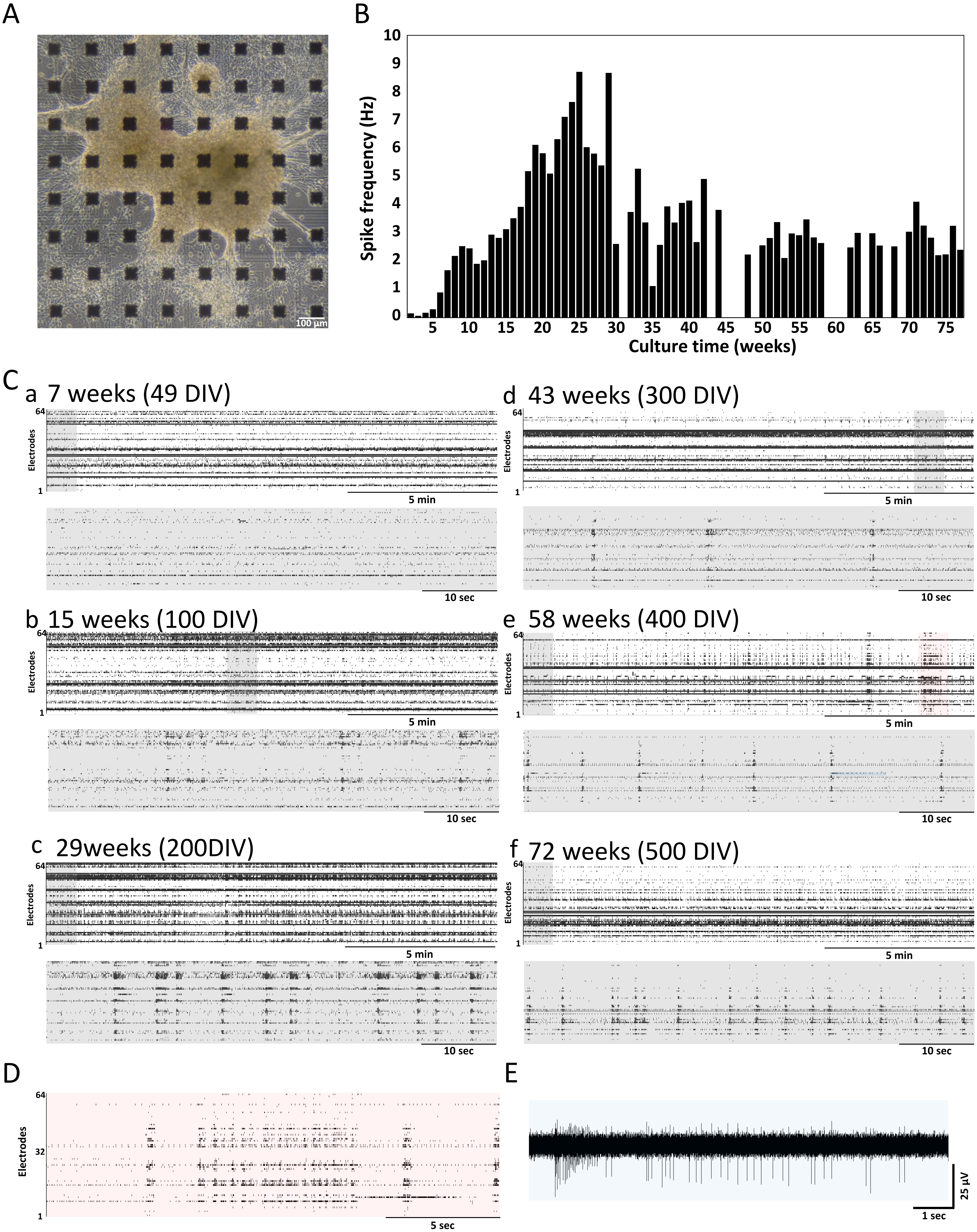

A
100 µm
B
Spike frequency (Hz)
Culture time (weeks)
0
1
2
3
4
5
6
7
8
9
10
5
10
15
20
25
30
35
40
45
50
55
60
65
70
75
C
a 7 weeks (49 DIV)
b 15 weeks (100 DIV)
c 29weeks (200DIV)
d 43 weeks (300 DIV)
e 58 weeks (400 DIV)
f 72 weeks (500 DIV)
Electrodes
64
1
5 min
10 sec
D
Electrodes
64
32
1
5 sec
E
25 µV
1 sec

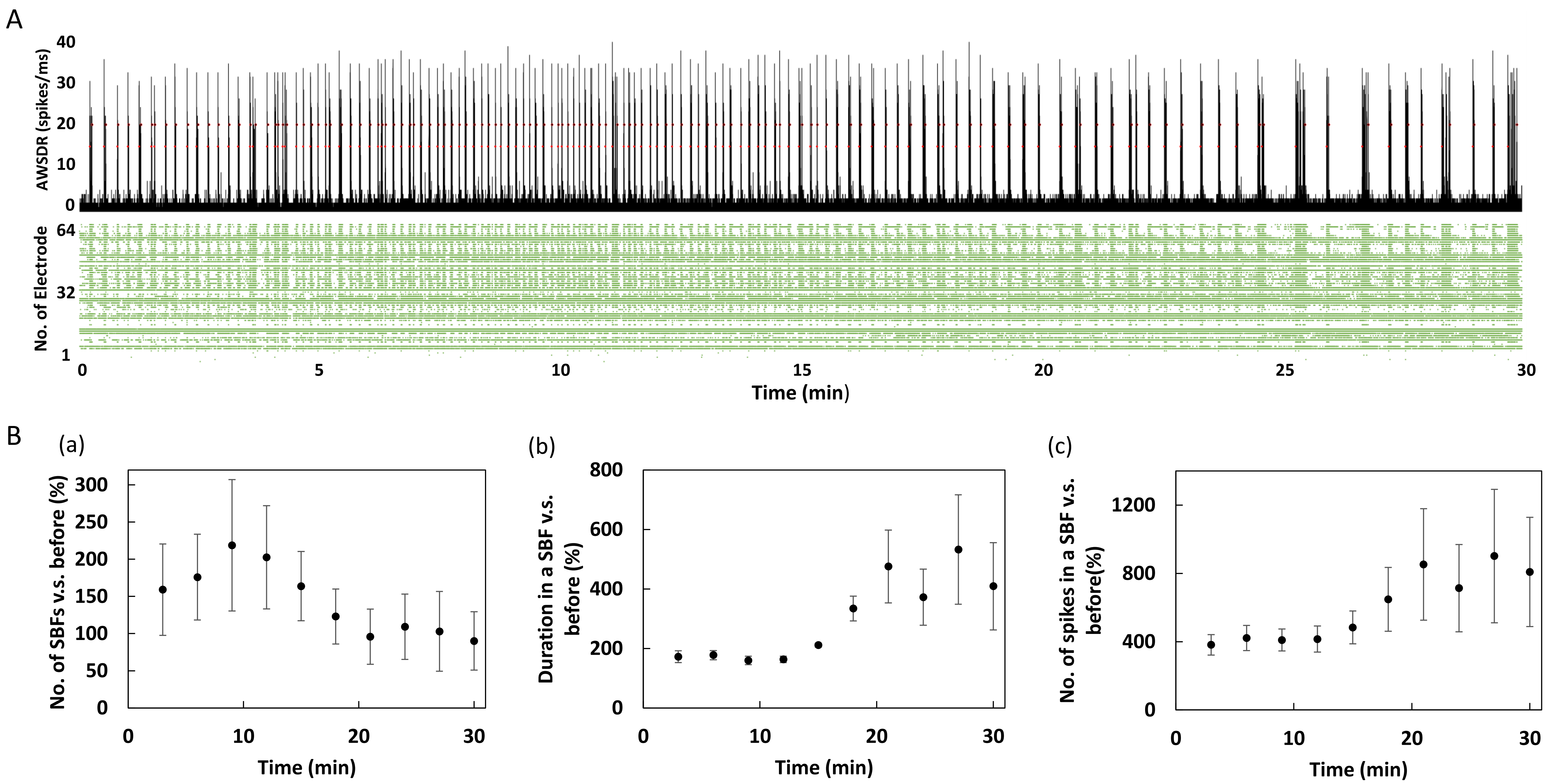

A
AWSDR (spikes/ms)
40
30
20
10
0
No. of Electrode
64
32
1
0
5
10
15
20
25
30
Time (min)
B
(a)
No. of SBFs v.s. before (%)
300
250
200
150
100
50
0
Time (min)
(b)
Duration in a SBF v.s. before (%)
800
600
400
200
Time (min)
(c)
No. of spikes in a SBF v.s. before(%)
1200
800
400
Time (min)

Supplementary Figure 1

Spontaneous firing recorded over 544 days. (A) Phase-contrast image of hiPSC-derived cortical neuronal networks on an MEA at 500 DIV. (B) Change in spike frequency per channel (average) over 72 weeks (after 30 weeks, some weekly recordings were missed). (C) 64-channel raster plots of spontaneous firing at (a) 7 WIV (49 DIV), (b) 15 WIV (100 DIV), (c) 29 WIV (200 DIV), (d) 43 WIV (300 DIV), (e) 58 WIV (400 DIV), and (f) 72 WIV (500 DIV). Upper shows raster plots for 15 min. Lower shows magnified raster plots for 50 s corresponding to the gray-shaded span in upper raster plots. (D) 64-ch raster plots of a SBF over 5 s in duration. This data is from the pink-shaded span of the raster plot in Figure S1-C-e (400 DIV). (E) Waveform of a super burst in a single neuron. This waveform is from the blue-shaded span of the raster plot in Figure S1-C-e (400 DIV).

Supplementary Figure 2

Time course of the spontaneous spiking response after bicuculline administration at 33–36 WIV. (A) The 64 electrode array-wide spike detection rate (spikes/ms) and raster plots for 30 min after bicuculline administration. Orange and red dots are the start and end points of a SBF, respectively. (B) Analysis of SBFs. Bin size = 3 min. (a) Change in number of SBFs relative to before administration (%). (b) Change in SBF duration. (c) Changes in number of spikes per SBF.

Supplementary Movie 1

The movie shows immunofluorescent images through the Z-axis of cultured hiPSC-derived cortical neurons at 300 DIV (height: 10 μm). Green: neuronal marker (β-tubulin III). Red: presynapse marker (synaptophysin).

Supplementary Movie2

Raw data of spikes at 20 WIV before drug administration, after 1 mM PTZ, and after 10 μM phenytoin.

Supplementary Movie2

Raw data of spikes at 20 WIV before drug administration, after 1 mM PTZ, and after 10 μM phenytoin.
